# Mechanistic insights into plasmid transfer inhibition in Enterobacterales by nucleoside analogues

**DOI:** 10.1038/s44259-026-00197-5

**Published:** 2026-04-14

**Authors:** Ilyas Alav, Ayesha Ashraf, Parisa Pordelkhaki, Michelle M. C. Buckner

**Affiliations:** 1 Department of Microbes, Infection and Microbiomes, School of Infection, Inflammation and Immunology, College of Medicine and Health, University of Birmingham, Birmingham, UK; 2 Present Address: Sir William Dunn School of Pathology, University of Oxford, Oxford, UK

**Keywords:** Microbiology, Molecular biology

## Abstract

Antimicrobial resistance (AMR) poses a major global health threat, with carbapenem-resistant and extended-spectrum β-lactamase (ESBL)-producing Enterobacterales causing widespread infections and deaths. Much of this resistance spreads through conjugative plasmids, autonomously replicating mobile genetic elements that transfer between bacteria and carry multiple AMR genes. Targeting plasmid conjugation could therefore help curb the spread of AMR. In this study, we tested whether clinically approved nucleoside analogues (NAs) inhibited the transfer of the GFP-tagged ESBL-encoding IncK plasmid pCT*gfp* in *Escherichia coli* and the carbapenemase-encoding IncF plasmid pKpQIL*gfp* in *Klebsiella pneumoniae* using flow cytometry. Alongside the known inhibitor azidothymidine (AZT), didanosine, stavudine, and trifluridine reduced plasmid conjugation in both species without affecting growth. Conversely, famciclovir and zalcitabine promoted pCT*gfp* conjugation in *E. coli*, while aciclovir and valaciclovir enhanced pKpQIL*gfp* conjugation in *K. pneumoniae*. Mechanistic studies showed that plasmid conjugation-promoting NAs altered intracellular ATP levels. RNA sequencing revealed that AZT downregulated the expression of genes linked to motility in *E. coli*. Genetic inactivation of motility in *E. coli* mirrored the decrease in pCT*gfp* conjugation, like AZT. In *K. pneumoniae*, AZT upregulated genes linked to DNA damage and the SOS response, but downregulated methionine biosynthesis and metabolism genes. The exogenous addition of zinc acetate to inhibit RecA or the end product of methionine metabolism, *S*-adenosyl-methionine, restored pKpQIL*gfp* conjugation in *K. pneumoniae*. Overall, our results indicated that existing NAs, including AZT, represent structural scaffolds for the development of potent conjugation inhibitors and highlight motility, DNA repair, and methionine metabolism as potential key factors in plasmid conjugation.

## Introduction

Antimicrobial resistance (AMR) in bacterial pathogens is a major global public health issue. Specifically, carbapenem-resistant and extended-spectrum β-lactamase (ESBL)-producing Enterobacterales are classified as critical priority pathogens by the World Health Organisation for research, development, and public health initiatives^1^. Within Enterobacterales, *Escherichia coli* and *Klebsiella pneumoniae* strains were estimated to be responsible for most human mortality attributed to antibiotic resistance in 2019^2^. Most AMR genes (ARGs) in *K. pneumoniae* and *E. coli* are found on mobile genetic elements, including conjugative plasmids that often carry multiple ARGs, resulting in multidrug resistance^3–5^. Conjugative plasmids can replicate and transfer autonomously, thereby facilitating the dissemination of AMR genes throughout bacterial populations^6,7^.

Several characteristics of conjugative plasmids enable their dissemination and persistence in bacterial populations. For example, the CTX-M-14 ESBL-encoding IncK plasmid pCT has a low fitness cost on its host, can persist in the presence or absence of antibiotic selective pressure, and is readily transmitted between bacteria^8,9^. This is also the case for carbapenemase-encoding IncF plasmids, such as pKpQIL and pCPE16_3, that transfer at high frequencies in *K. pneumoniae* strains^10,11^. Plasmid-mediated transmission of ARGs occurs in clinical environments, often resulting in hospital outbreaks of carbapenemase-producing Enterobacterales (CPE). For example, in a retrospective study of public Singaporean hospitals involving 779 patients and 1215 CPE isolates, 50% of CPE dissemination was due to plasmid-mediated transmission of ARGs^12^. Similar findings have been reported across the world, such as in Thailand, the US, and the UK^13–15^, highlighting the global problem of plasmid-mediated spread of ARGs. Notably, the otherwise promising β-lactam/β-lactamase inhibitor combinations, such as ceftazidime/avibactam and piperacillin/tazobactam, have been shown to be ineffective against *K. pneumoniae* strains carrying variants of pKpQIL plasmids^16,17^. Therefore, there is an urgent unmet need for alternative strategies to tackle AMR, such as targeting AMR plasmids.

One approach to target AMR plasmids is the development and use of anti-plasmid compounds^18^. These may alter the stability of AMR plasmids, entirely remove the plasmid (plasmid curing), or impair the dissemination of AMR genes by inhibiting horizontal gene transfer, such as bacterial conjugation^19^. A range of compounds exhibiting variable anti-plasmid activity have been identified, such as unsaturated fatty acids, natural products, and clinically approved drugs^20–22^. To date, the use of anti-plasmid compounds is in early stages with none having been approved for clinical use. Previous work by our group identified two nucleoside analogues (NAs) as novel anti-plasmid agents: abacavir and azidothymidine (AZT). In particular, AZT significantly reduced the transmission of pCT*gfp* in *E. coli* and pKpQIL*gfp* in *K. pneumoniae* at below peak serum concentrations, without affecting bacterial growth^21^.

In this study, we explored whether a range of clinically approved nucleoside analogues modulated plasmid transmission in *E. coli* and *K. pneumoniae*. We found that famciclovir and zalcitabine promoted the conjugation frequency of the ESBL-encoding IncK plasmid pCT in *Escherichia coli*, and aciclovir and valaciclovir promoted the conjugation frequency of the carbapenemase-encoding IncF plasmid pKpQIL in *Klebsiella pneumoniae*. We identified three additional nucleoside analogues, didanosine, stavudine, and trifluridine, that reduced the conjugation frequencies of pCT in *E. coli* and pKpQIL in *K. pneumoniae*. As AZT showed the most potent activity, we explored the mechanism by which AZT reduced plasmid conjugation in *E. coli* and *K. pneumo*niae. RNA sequencing revealed that AZT significantly reduced the expression of motility genes in *E. coli*, and DNA repair pathway genes and methionine metabolism genes in *K. pneumoniae*. In phenotypic testing, this translated to a reduction in pCT*gfp* conjugation frequency in *E. coli* through inhibition of motility. In *K. pneumoniae*, addition of zinc acetate, which reversed *recA* expression, or *S*-adenosyl-methionine, which restored depleted levels, reversed AZT-mediated reduction in pKpQIL*gfp* conjugation frequency. Our work highlights the potential use of clinically approved NAs as structural scaffolds for the development of novel plasmid transmission inhibitors and sheds light onto the complex mechanism by which AZT reduces plasmid conjugation.

## Results

### Screening of clinically approved nucleoside analogues for plasmid transmission inhibitors

To identify additional nucleoside analogues (NAs) as potential plasmid transmission inhibitors, 14 clinically approved NAs were screened using flow cytometry. Firstly, the minimum inhibitory concentration (MIC) of all NAs for the *E. coli* EC958 and *K. pneumoniae* Ecl8 strains was determined. The MICs of almost all NAs were greater than 256 µg/mL, except for azidothymidine (AZT) and didanosine, which had MICs of 2 and 128 µg/mL, respectively (Supplementary Table 1). Accordingly, all NAs, except AZT, were tested at 1, 10 and 100 µg/mL for the initial flow cytometry screening of plasmid inhibition activity. Previously, we found AZT to be effective at reducing plasmid transmission at concentrations as low as 0.008 µg/mL^21^, therefore, this concentration was used throughout. As expected, 0.008 µg/mL AZT significantly reduced transmission of pCT*gfp* in *E. coli* and pKpQIL*gfp* in *K. pneumoniae*. Most of the NAs at the tested concentrations did not significantly affect pCT*gfp* and pKpQIL*gfp* transmission (Supplementary Fig. 1). Notably, didanosine reduced the transmission of both pCT*gfp* in *E. coli* and pKpQIL*gfp* in *K. pneumoniae* at 10 and 100 µg/mL, and stavudine and trifluridine reduced plasmid transmission at 100 µg/mL (Supplementary Fig. 1). Conversely, 10 and 100 µg/mL aciclovir and 1, 10 and 100 µg/mL valaciclovir significantly increased pKpQIL*gfp* transmission in *K. pneumoniae* (Supplementary Fig. 1). In *E. coli*, 1, 10 or 100 µg/mL famciclovir or zalcitabine significantly increased pCT*gfp* transmission (Supplementary Fig. 1). Therefore, from the initial screen, didanosine, stavudine, and trifluridine were selected for further investigation to narrow down the lowest concentration at which plasmid transmission was inhibited. Aciclovir, famciclovir, valaciclovir, and zalcitabine were also selected for further investigation as they significantly increased plasmid transmission. The chemical structures of the nucleoside analogues that reduced and promoted plasmid transfer are illustrated in Supplementary Fig. 2.

### Didanosine, stavudine, and trifluridine reduced the conjugation of pCT*gfp* and pKpQIL*gfp*

**Fig. 1:**
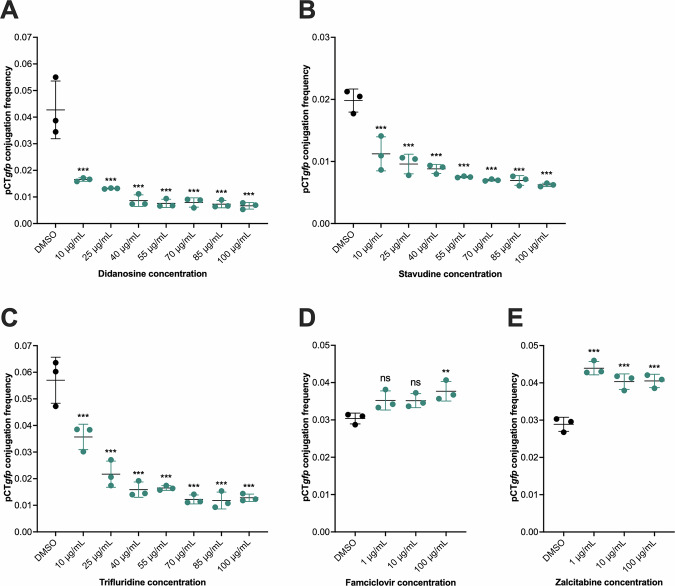
The effect of selected nucleoside analogues (NAs) from the initial screen on pCT*gfp* conjugation in *Escherichia coli* EC958. The conjugation frequency (CF) of pCT*gfp* from EC24 (*E. coli* EC958/pCT*gfp*) to EC25 (*E. coli* EC958 *mCherry*) in the presence of **A** didanosine, **B** stavudine, **C** trifluridine, **D** zalcitabine, and **E** famciclovir. The CF of pCT*gfp* was determined as the number of transconjugants divided by the number of recipient cells. Data presented are the mean ± standard deviation of three independent experiments, each consisting of four biological replicates. The CF of pCT*gfp* treated with DMSO was compared to those treated with the NAs using one-way ANOVA, followed by the Dunnett’s test to correct for multiple comparisons. Significantly different results are indicated with * (*P* ≤ 0.05) ** (*P* ≤ 0.01) or *** (*P* < 0.001). ns not significant.

To determine the lowest concentration of didanosine, stavudine, and trifluridine that reduced plasmid conjugation, a range of concentrations between 10 and 100 µg/mL was tested (10, 25, 40, 55, 70, 85, and 100 µg/mL). In *E. coli*, didanosine significantly reduced the conjugation frequency (CF) of pCT*gfp* starting at 10 µg/mL, with slightly further reductions at 25 µg/mL and above compared to DMSO control (Fig. 1). For stavudine and trifluridine, 10 µg/mL was also the lowest concentration that significantly reduced the CF of pCT*gfp* in *E. coli* (Fig. 1). For famciclovir, 100 µg/mL significantly increased the CF of pCT*gfp*, whilst 1, 10 and 100 µg/mL zalcitabine significantly increased the CF of pCT*gfp* (Fig. 1). The growth and mean generation times of the *E. coli* strains EC24 and EC25 grown in LB broth supplemented with 0.008 µg/mL azidothymidine, 10 µg/mL didanosine, 25 µg/mL stavudine, 25 µg/mL trifluridine, 100 µg/mL zalcitabine or 100 µg/mL famciclovir were not significantly different to the DMSO vehicle control (Supplementary Figs. 3 and 5). Therefore, these concentrations, which impacted CF without affecting growth, were selected for *E. coli* mechanism of action investigations.

**Fig. 2:**
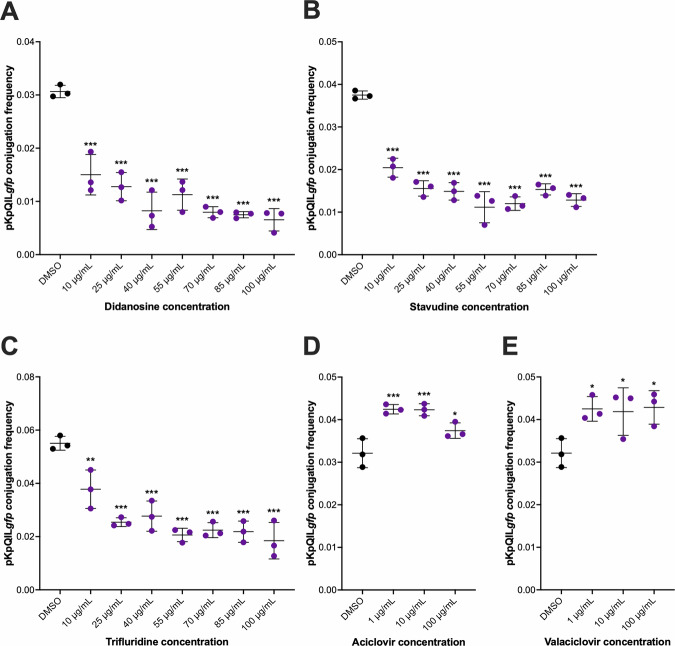
The effect of selected nucleoside analogues (NAs) from the initial screen on pKpQIL*gfp* conjugation in *Klebsiella pneumoniae* Ecl8. The conjugation frequency (CF) of pKpQIL*gfp* from KP19 (*K. pneumoniae* Ecl8/pKpQIL*gfp*) to KP18 (*K. pneumoniae* Ecl8 *mCherry*) in the presence of **A** didanosine, **B** stavudine, **C** trifluridine, **D** valaciclovir, and **E** aciclovir. The CF of pKpQIL*gfp* was determined as the number of transconjugants divided by the number of recipient cells. Data presented are the mean ± standard deviation of three independent experiments, each consisting of four biological replicates. The CF of pKpQIL*gfp* treated with DMSO was compared to those treated with the NAs using one-way ANOVA, followed by Dunnett’s test to correct for multiple comparisons. Significantly different results are indicated with * (*P* ≤ 0.05) ** (*P* ≤ 0.01), or *** (*P* < 0.001). ns, not significant.

In *K. pneumoniae*, 10 µg/mL didanosine, stavudine, and trifluridine significantly reduced the CF of pKpQIL*gfp* compared to DMSO control (Fig. 2). However, since 10 µg/mL stavudine had no effect on pKpQIL*gfp* transmission in the preliminary screen (Supplementary Fig. 1), 25 µg/mL was selected for downstream experiments. At 1, 10 and 100 µg/mL, both aciclovir or valaciclovir significantly increased the CF of pKpQIL*gfp* compared to the DMSO control (Fig. 2). The growth and mean generation times of the *K. pneumoniae* strains KP18 and KP19 grown in LB broth supplemented with 0.008 µg/mL azidothymidine, 10 µg/mL didanosine, 25 µg/mL stavudine, 25 µg/mL trifluridine, 100 µg/mL valaciclovir or 100 µg/mL aciclovir, were not significantly different to DMSO vehicle control (Supplementary Figs. 3 and 5), indicating no impact on growth. Therefore, these concentrations were selected to be used in the mechanism of action investigations for *K. pneumoniae*.

### Impact of nucleoside analogues on bacterial physiology

**Fig. 3:**
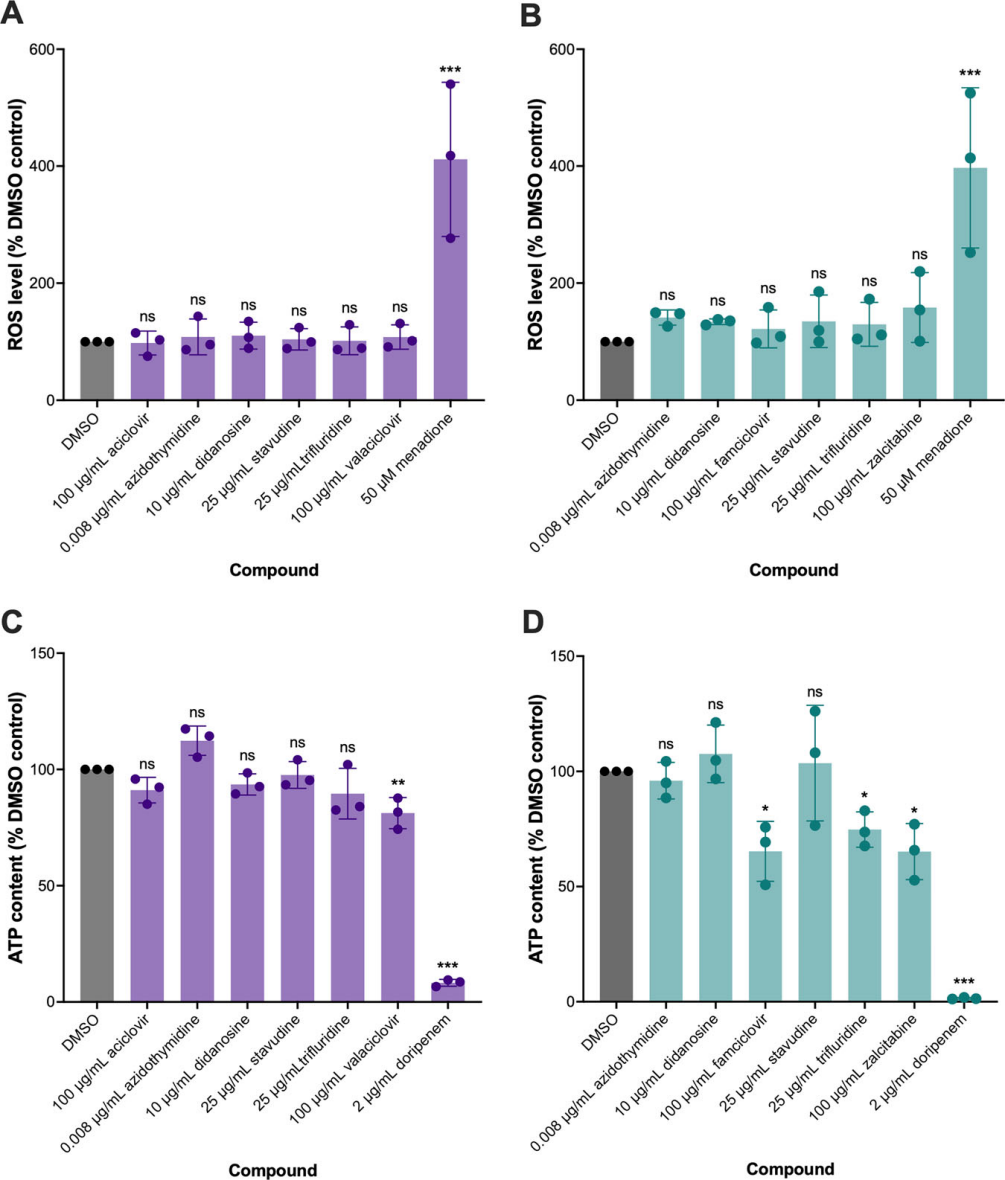
The effect of nucleoside analogues (NAs) on reactive oxygen species levels and ATP content in *Escherichia coli* EC958/pCT*gfp* and *Klebsiella pneumoniae* Ecl8/pKpQIL*gfp.* The level of reactive oxygen species was measured by ROS-Glo H_2_O_2_ Assay Kit in **A** *K. pneumoniae* Ecl8/pKpQIL*gfp* and **B** *E. coli* EC958/pCT*gfp*, following treatment with the indicated concentrations of NAs or 50 µM menadione as positive control for 4 h. Bacterial cells treated with vehicle control (1% DMSO) were used as negative control. The total intracellular ATP content was measured by BacTiter-Glo Microbial Cell Viability Assay Kit in **C** *E. coli* EC958/pCT*gfp* and **D** *K. pneumoniae* Ecl8/pKpQIL*gfp*, following treatment with the indicated concentrations of NAs or 2 µg/mL doripenem as a positive control for 4 h. Bacterial cells treated with vehicle control (1% DMSO) were used as a negative control. For both assays, the data presented are the mean ± standard deviation of ROS levels or ATP content in treated bacteria relative to the DMSO control, tested in three independent experiments, each comprising a biological replicate tested in triplicate. Statistical significance was determined by comparing relative ROS levels or ATP content in treated bacteria to the DMSO control by one-way ANOVA, followed by Dunnett’s test to correct for multiple comparisons. Significantly different results are indicated with * (*P* ≤ 0.05), ** (*P* < 0.01), or *** (*P* < 0.001). ns, not significant.

Compounds that affect plasmid conjugation have been reported to influence various aspects of bacterial physiology, including membrane permeability and potential, reactive oxygen species (ROS) generation, and ATP synthesis^23^. To that end, we investigated the impact of NAs on some of these processes. Since the donor strains in our assays carried conjugative AMR plasmids tagged with GFP, luminescence-based assays were used to measure ROS levels and ATP content, as commercially available dyes for ROS or ATP detection have overlapping wavelengths with GFP. The bacterial strains were treated with the NAs for the same timeframe and conditions as per the conjugation assays. The treatment of the donor *E. coli* strain EC24 and the donor *K. pneumoniae* strain KP19 with the NAs for 4 h did not significantly affect ROS levels, whilst the positive control of 50 µM menadione significantly increased ROS levels, compared to DMSO control (Fig. 3A, B). Conjugative plasmid transmission is mediated by type IV secretion systems, which require ATP to function^24^. Therefore, we investigated whether any of the NAs affected ATP generation in the donor strains. As a positive control, 2 µg/mL doripenem was used as it is bactericidal and caused a severe reduction in ATP content due to cell death (Fig. 3B, D). In *K. pneumoniae* Ecl8/pKpQIL*gfp*, most of the NAs did not significantly impact ATP content following treatment for 4 h. However, 100 µg/mL valaciclovir caused a modest yet significant reduction in ATP content (Fig. 3C). In *E. coli* EC958/pCT*gfp*, 100 µg/mL famciclovir, 25 µg/mL trifluridine, and 100 µg/mL zalcitabine significantly reduced ATP content after 4 h (Fig. 3D).

**Fig. 4:**
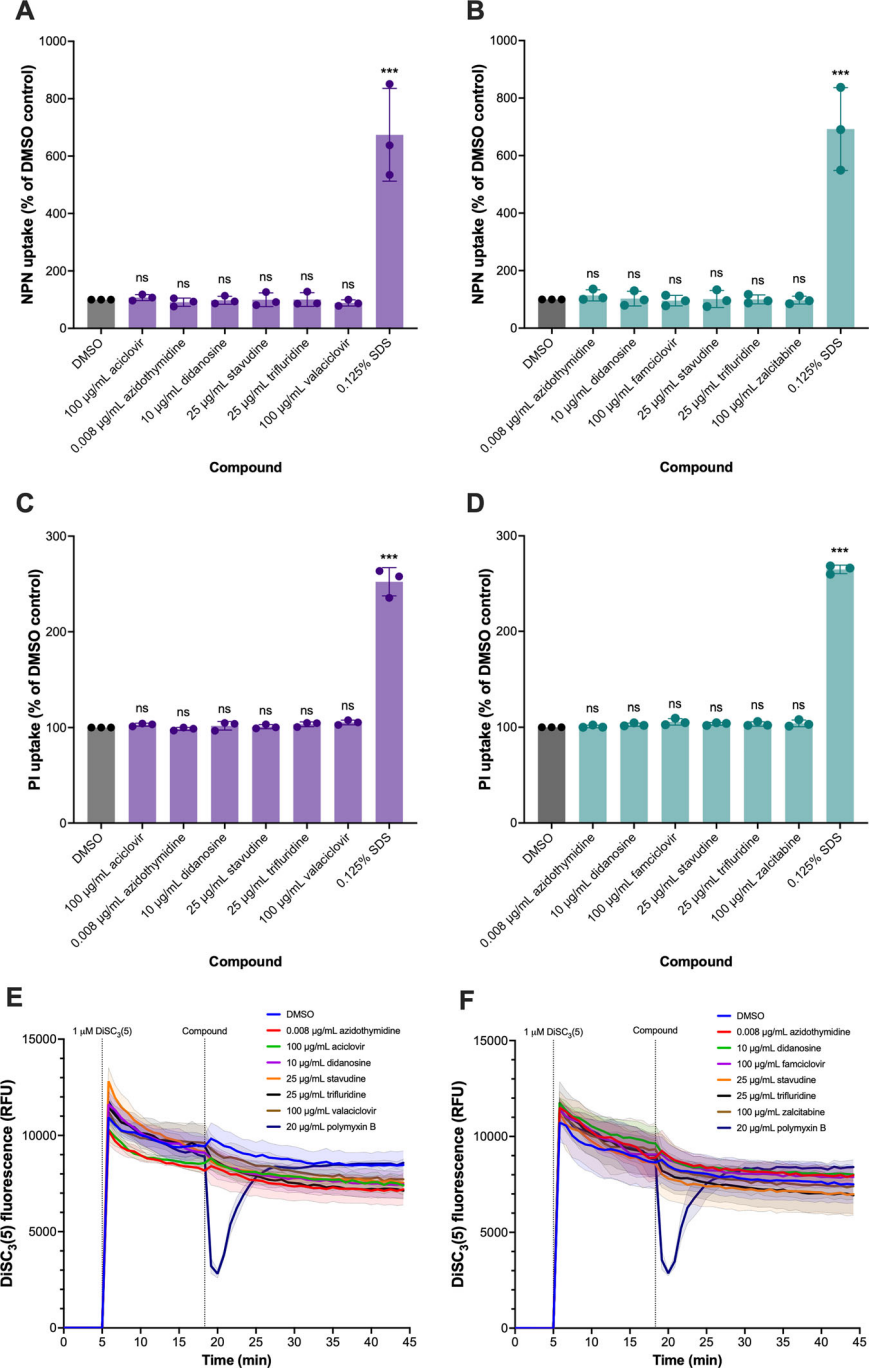
The effect of nucleoside analogues on membrane permeability and potential in *Escherichia coli* EC958/pCT*gfp* and *Klebsiella pneumoniae* Ecl8/pKpQIL*gfp.* The effect of nucleoside analogues on outer membrane permeability was determined by measuring the uptake of the hydrophobic dye *N*-phenyl-1-naphthylamine (NPN) in **A** *K. pneumoniae* Ecl8/pKpQIL*gfp* and **B** *E. coli* EC958/pCT*gfp*, following treatment with the indicated concentrations of NAs or 0.125% sodium dodecyl sulphate (SDS) as a positive control for 4 h. The effect of nucleoside analogues on inner membrane permeability was determined by measuring uptake of the intercalating dye propidium iodide in **C** *K. pneumoniae* Ecl8/pKpQIL*gfp* and **D** *E. coli* EC958/pCT*gfp*, following treatment with the indicated concentrations of NAs or 0.125% sodium dodecyl sulphate (SDS) as a positive control for 4 h. The effect of nucleoside analogues on membrane potential was determined using the voltage-sensitive dye DiSC_3_(5) (3,3’-dipropylthiadicarbocyanine iodide) in **E** *K. pneumoniae* Ecl8/pKpQIL*gfp* and **F** *E. coli* EC958/pCT*gfp*. DiSC_3_(5) was added to bacterial cells and allowed to quench, followed by the addition of indicated concentrations of NAs or 20 µg/mL polymyxin B as a positive control. For all assays, bacterial cells treated with vehicle control (1% DMSO) were used as a negative control. Data presented are the mean ± standard deviation of three biological replicates, each tested in triplicate, on independent occasions. Statistical significance for the NPN and propidium iodide uptake assays was determined by comparing the mean of the DMSO control to the treatment groups using one-way ANOVA, followed by Dunnett’s test to correct for multiple comparisons. Significantly different results are indicated with *** (*P* < 0.001). ns, not significant.

Next, we explored the impact of the NAs on membrane permeability and potential. To measure outer membrane permeability, we used the dye NPN (*N*-phenyl-1-naphthylamine), a hydrophobic fluorescent dye that is normally excluded by the intact outer membrane but displays increased fluorescence upon partitioning into compromised membranes. The detergent sodium dodecyl sulphate (SDS) was used as a positive control, as it is a well-known membrane permeabilizing agent. As expected, 0.125% SDS significantly increased outer membrane permeability in both *E. coli*/pCT*gfp* and *K. pneumoniae*/pKpQIL*gfp* (Fig. 4A, B). However, none of the NAs significantly affected NPN fluorescence in both species, indicating a lack of outer membrane permeabilising effects by the NAs (Fig. 4A, B). To measure inner membrane permeability, we used propidium iodide (PI), a fluorescent dye that cannot penetrate intact inner membranes but fluoresces highly once it intercalates with DNA. As expected, 0.125% SDS significantly increased PI fluorescence (Fig. 4C, D). Similarly to the NPN uptake assay, none of the NAs significantly affected PI fluorescence, indicating a lack of membrane-permeabilising effects in both *E. coli* and *K. pneumoniae*. Lastly, we assessed the effect of NAs on bacterial membrane potential using the voltage-sensitive dye DiSC_3_(5) (3,3’-dipropylthiadicarbocyanine iodide), which accumulates on hyperpolarised membranes and is translocated into lipid bilayers. As a positive control, we used polymyxin B, which disrupts the outer membrane and causes rapid depolarisation, indicated by a transient reduction in DiSC_3_(5) fluorescence in both *E. coli* and *K. pneumoniae* (Fig. 4E, F). At the tested concentrations, none of the NAs had an observable effect on the membrane potential of *E. coli* and *K. pneumoniae* (Fig. 4E, F). Together, these results highlighted that the NAs did not affect the membrane physiology of *E. coli* and *K. pneumoniae*.

### Exploring the effects of azidothymidine (AZT) on bacterial gene expression

In order to provide more in-depth insight into the mechanism of action, we used RNA sequencing to assess how the most promising NA, AZT, affected the transcription of *E. coli* EC958/pCT*gfp* and *K. pneumoniae* Ecl8/pKpQIL*gfp*. RNA sequencing was carried out for both strains (*n* = 4) grown in LB broth supplemented with 0.008 µg/mL AZT or an equal volume of DMSO as a vehicle control. Differentially expressed genes were determined by comparing DMSO-treated samples to the AZT-treated samples for both strains. In both species, most of the significantly upregulated genes were associated with DNA damage repair pathways and the SOS response. However, there were major differences in the type of downregulated genes between the two species.

### Azidothymidine reduces pCT*gfp* conjugation in *Escherichia coli* EC958 by impairing motility

**Fig. 5:**
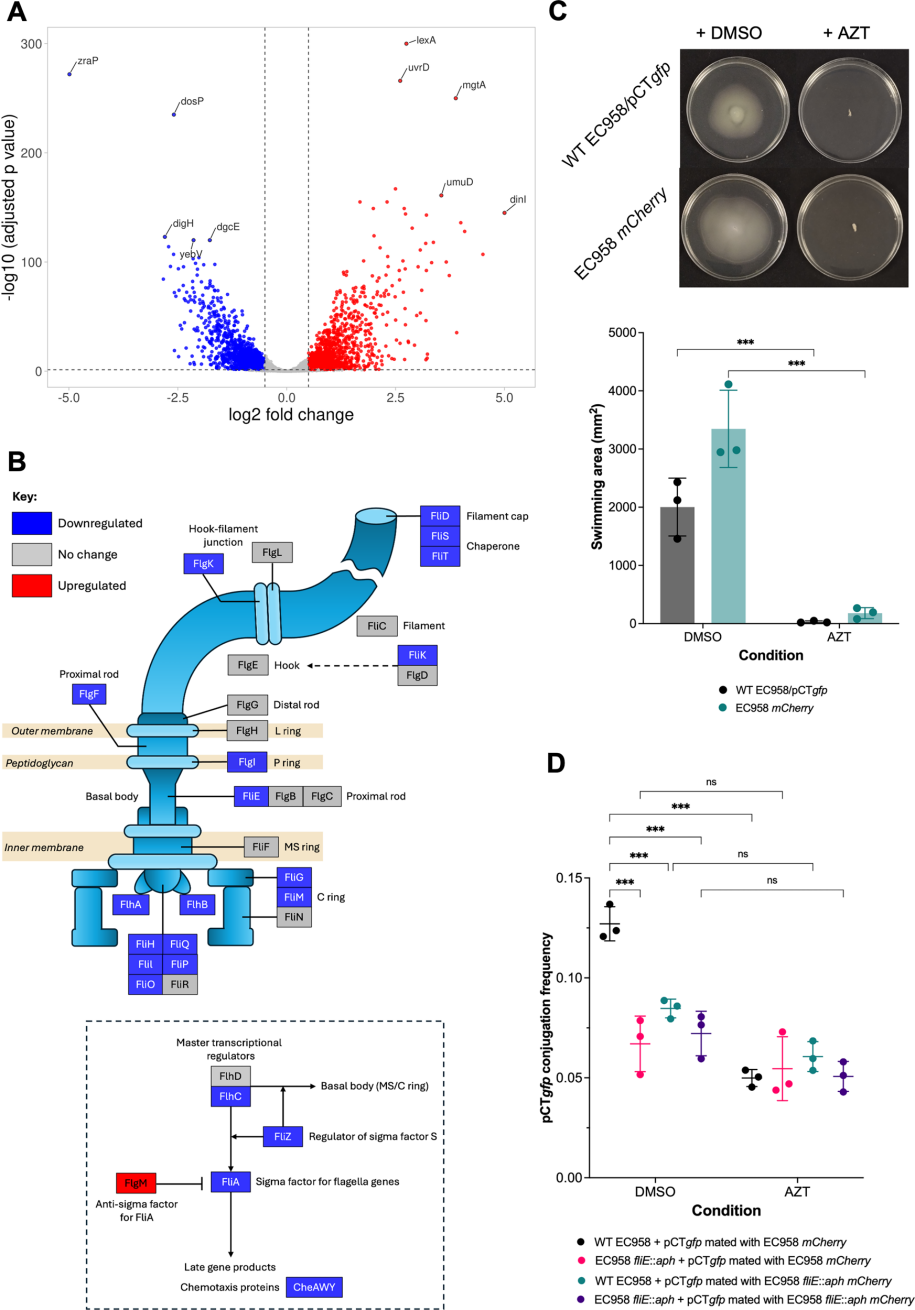
Investigating the mechanism of action of azidothymidine on pCT*gfp* conjugation in *Escherichia coli* EC958. **A** Volcano plot of differentially expressed genes in *E. coli* EC958/pCT*gfp* treated with 0.008 µg/mL azidothymidine (AZT) compared to DMSO control (*n* = 4). Dots in blue and red indicate significantly downregulated and upregulated genes, respectively. The log_2_ fold-change cut-off and adjusted p-value cut-off were 0.5 and 0.05, respectively. **B** Schematic diagram of the flagellum labelled with the structural proteins and the transcriptional regulation of the flagella operon (in dashed box), coloured according to whether the corresponding genes are significantly downregulated or upregulated. **C** Swimming motility of *E. coli* EC958/pCT*gfp* and EC958 *mCherry* on 0.3% agar supplemented with 0.008 µg/mL AZT or an equal volume of DMSO as a control. Plates were incubated at 37 °C for 18 h. Swimming motility was determined by measuring the diameter and calculating the area of the swimming zone. Representative images of swimming motility by EC958/pCT*gfp* and EC958 *mCherry* treated with AZT or DMSO (top). Swimming area of EC958/pCT*gfp* and EC958 *mCherry* treated with 0.008 µg/mL AZT or DMSO (bottom). Data presented are the mean ± standard deviation of three independent experiments, each consisting of three biological replicates. The mean swimming area of EC958/pCT*gfp* and EC958 *mCherry* treated with 0.008 µg/mL AZT was compared to that of DMSO treatment using an unpaired two-tailed *t*-test. Significantly different results are indicated with *** (*P* < 0.001). **D** Flow cytometry-based measurement of pCT*gfp* conjugation frequency (CF) from the motile donor EC24 (WT EC958/pCT*gfp*) or the non-motile donor EC83 (*fliE*::*aph* EC958/pCT*gfp*) to the motile recipient EC25 (EC958 *mCherry*) or the non-motile recipient EC85 (*fliE*::*aph* EC958 *mCherry*) treated with 0.008 µg/mL AZT or an equal volume of DMSO. The CF of pCT*gfp* was determined as the number of transconjugant cells divided by the number of recipient cells. Data presented are the mean ± standard deviation of three independent experiments, each consisting of four biological replicates. The indicated comparisons were carried out using one-way ANOVA, followed by the Holm-Šídák test to correct for multiple comparisons. Significantly different results are indicated with *** (*P* < 0.001). ns, not significant.

In *E. coli* EC958/pCT*gfp*, the most downregulated genes were *zraP* and *dosP*, encoding the zinc resistance-associated protein ZraP and the oxygen-sensor protein DosP, respectively. Other downregulated genes included *dgcE* (encodes diguanylate cyclase), *digH* (encodes glycosyl hydrolase), and *yebV* (encodes DUF1480 family protein) (Supplementary Data 1). Of the genes on pCT*gfp*, the most upregulated gene, was HXG14_RS00170, encoding for the post-segregation killing protein PndC, and the most downregulated gene was HXG14_RS00465, encoding for the type IV pilus major pilin (Supplementary Data 1). Notably, 27 genes within flagellar operons, including genes encoding the flagellar basal body, hook, and filament, were significantly downregulated. Genes encoding the master flagella transcriptional regulator FlhC and the downstream flagella-specific sigma factor FliA were also significantly downregulated. The anti-sigma factor for FliA, FlgM, was significantly upregulated (Fig. 5B). These data strongly suggested that AZT inhibited flagella biosynthesis and motility in *E. coli* EC958. Therefore, we tested the effect of AZT on swimming motility in both the donor EC958 carrying pCT*gfp* and the recipient EC958 *mCherry* strains. Supplementing swimming agar with 0.008 µg/mL AZT significantly impaired swimming motility compared to DMSO. The swimming area decreased from approximately 2000–3000 mm^2^ to near negligible across both donor and recipient *E. coli* EC958 strains (Fig. 5C). The reduction in swimming motility was not due to growth inhibition as 0.008 µg/mL azidothymidine had no effect on the growth of *E. coli* EC958/pCT*gfp* or *E. coli* EC958 mCherry (Supplementary Fig. 3). This indicated that the altered expression of motility genes observed from the RNA sequencing results translated to impaired motility.

We hypothesised that the loss of motility mediated by AZT could reduce the likelihood of donor and recipient cells encountering and interacting to facilitate plasmid transfer. To test this hypothesis, we inactivated one of the significantly downregulated flagella genes, *fliE*, encoding the flagellar hook-basal body complex protein FliE, in both the donor EC958/pCT*gfp* and the recipient EC958 *mCherry* strains. This inactivation of *fliE* in both the donor and recipient strains completely abrogated swimming motility in both the donor and recipient EC958 strains, as expected (Supplementary Fig. 5). Firstly, we wanted to ascertain whether loss of motility in the donor or recipient had a greater impact on pCT*gfp* conjugation compared to 0.008 μg/mL AZT. Secondly, we wanted to find out if AZT exerted any further influence on pCT*gfp* conjugation when motility was already impaired. Compared with the fully motile strains, the CF of pCT*gfp* was significantly reduced when motility was impaired in the *E. coli* EC958 donor, recipient or both (Fig. 5D). A similar reduction in CF was observed in the fully motile strains treated with AZT compared with the DMSO control (Fig. 5D). Treatment of strain pairs where one or both strains were non-motile with AZT did not significantly further reduce the CF pCT*gfp,* supporting the hypothesis that AZT reduces conjugation in *E. coli* by limiting bacterial motility.

### Azidothymidine reduces pKpQIL*gfp* conjugation in *Klebsiella pneumoniae* Ecl8 partly by altering the expression of the DNA repair pathway and methionine metabolism genes

**Fig. 6:**
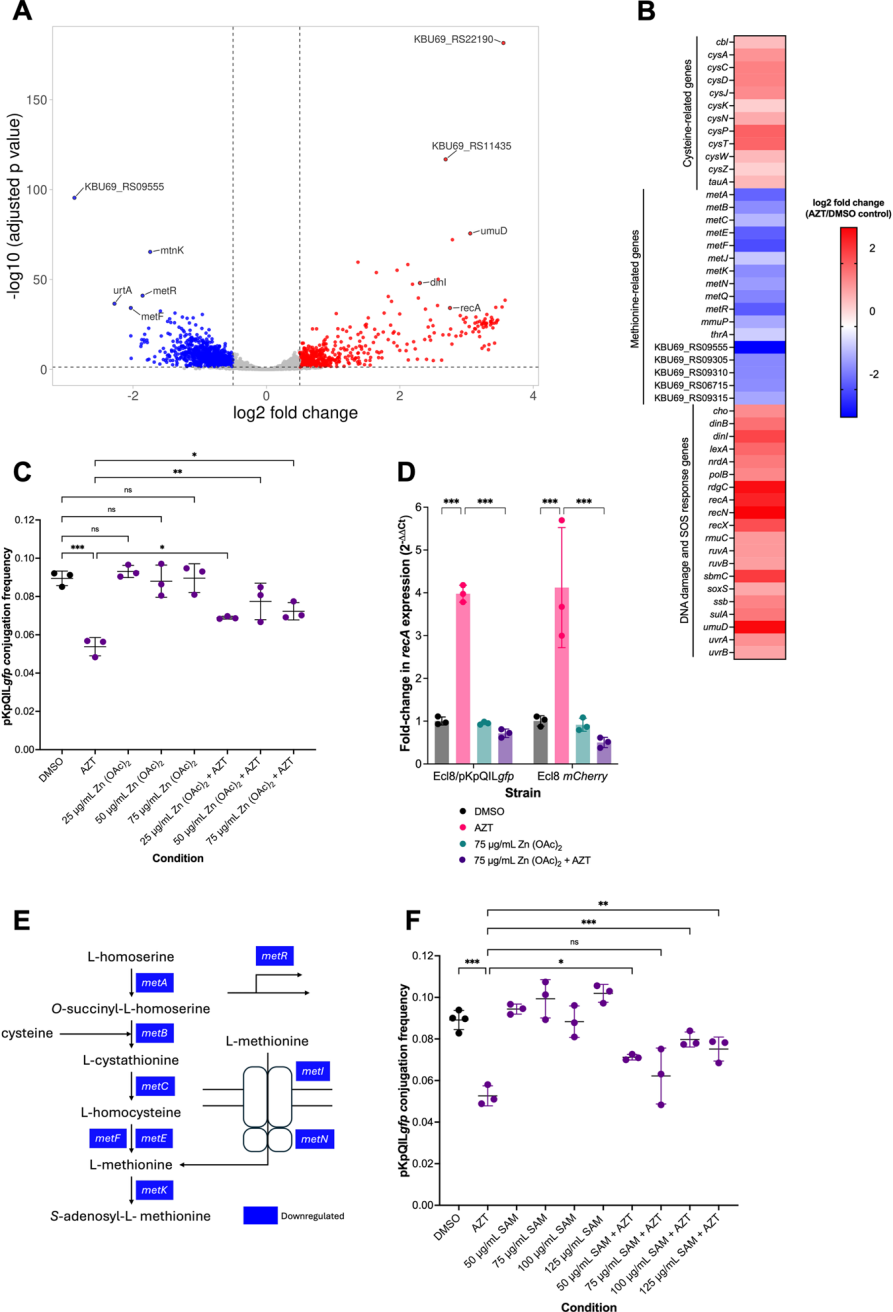
Investigating the mechanism of action of azidothymidine on pKpQILgfp conjugation frequency in Klebsiella pneumoniae Ecl8. **A** Volcano plot of differentially expressed genes in *K. pneumoniae* Ecl8/pKpQIL*gfp* treated with 0.008 µg/mL azidothymidine (AZT) compared to DMSO control (*n* = 4). Dots in blue and red indicate significantly downregulated and upregulated genes, respectively. The log_2_ fold-change cut-off and adjusted p-value cut-off was 0.5 and 0.05, respectively. **B** Selected differentially expressed genes involved in DNA repair and SOS response pathways, methionine biosynthesis, salvage, and transport, and cysteine metabolism. **C** Flow cytometry-based measurement of pKpQIL*gfp* conjugation frequency (CF) from KP19 (*K. pneumoniae* Ecl8/pKpQIL*gfp*) to KP18 (*K. pneumoniae* Ecl8 *mCherry*) treated with 0.008 µg/mL azidothymidine (AZT), zinc acetate (Zn (OAC)_2_), combination of both, or an equal volume of DMSO as control. The CF of pKpQIL*gfp* calculated as the number of transconjugant cells divided by the number of recipient cells. Data presented are the mean ± standard deviation of three independent experiments, each consisting of four biological replicates. The indicated comparisons were carried out using one-way ANOVA, followed by the Holm-Šídák test to correct for multiple comparisons. Significantly different results are indicated with * (*P* ≤ 0.05), ** (*P* < 0.01) or *** (*P* < 0.001). ns, not significant. **D** Fold-change in *recA* expression relative to *rpoD* in *K. pneumoniae* Ecl8 *mCherry* and *K. pneumoniae* Ecl8/pKpQIL*gfp* following treatment with 0.008 µg/mL AZT, 75 µg/mL Zn (OAC)_2_, or 0.008 µg/mL AZT and 75 µg/mL Zn (OAC)_2_, compared to DMSO vehicle control. The data presented are the mean ± standard deviations from three biological replicates tested in triplicate. Statistical significance was determined using one-way ANOVA, followed by the Holm-Šídák test to correct for multiple comparisons. Significantly different results are indicated with *** (*P* ≤ 0.001). ns, not significant. **E** A simplified methionine biosynthesis and transport pathway of *K. pneumoniae* annotated with the major downregulated genes induced by AZT treatment. *metR* encodes MetR, the positive activator of *metA* and *metE* genes. *metI* and *metN* encode components of the methionine import system. **F** Flow cytometry-based measurement of pKpQIL*gfp* CF from KP19 (*K. pneumoniae* Ecl8/pKpQIL*gfp*) to KP18 (*K. pneumoniae* Ecl8 *mCherry*) treated with 0.008 µg/mL AZT, *S*-adenosylmethionine (*S*AM), a combination of both, or an equal volume of DMSO as a control. Data presented are the mean ± standard deviation of three independent experiments, each consisting of four biological replicates. The indicated comparisons were carried out using one-way ANOVA, followed by the Holm-Šídák test to correct for multiple comparisons. Significantly different results are indicated with * (*P* ≤ 0.05), ** (*P* < 0.01), or *** (*P* < 0.001). ns, not significant.

In *K. pneumoniae* Ecl8/pKpQIL*gfp*, the RNA sequencing data showed significant upregulation of several genes involved in DNA damage response (Fig. 6A, B), such as *recN*, *dinI*, and *recA*, the latter encoding RecA, a key sensor of DNA damage and a regulator of the SOS response. Conjugation involves the transfer of a conjugative plasmid to a recipient cell in the form of a single-stranded DNA molecule. Under typical circumstances, the presence of such single-stranded DNA would activate RecA to trigger the SOS response, a cellular stress mechanism that can be detrimental to the cell^25^. Hence, RecA is inhibited by the product of the plasmid-encoded *psiB* gene to ensure a stable and controlled genetic exchange^26^. However, we did not find *psiB* to be differentially expressed. Therefore, we hypothesised that upregulation of *recA* expression induced by AZT was impeding efficient plasmid transmission in *K. pneumoniae*. A previous study showed that RecA expression in *E. coli* increased upon AZT exposure, which was reversed with the addition of zinc acetate^27^. We tested whether the AZT-mediated reduction in pKpQIL*gfp* CF was due to upregulation of *recA* expression, which could be reversed by the addition of zinc acetate. Therefore, we tested the effects of 0.008 µg/mL AZT combined with 25, 50, or 75 µg/mL of zinc acetate on the CF of pKpQIL*gfp* using flow cytometry. The combination of AZT with 25, 50 or 75 µg/mL zinc acetate significantly restored the CF of pKpQIL*gfp* compared to AZT treatment (Fig. 6C). To ascertain whether the effect of zinc acetate was mediated through downregulation of *recA* expression, RT-qPCR was carried out for both the donor *K. pneumoniae* Ecl8/pKpQIL*gfp* and the recipient *K. pneumoniae* Ecl8 *mCherry* strains. In agreement with the RNA sequencing data (Fig. 6B), exposure to 0.008 µg/mL AZT significantly increased *recA* expression by 4-fold in both the donor and recipient strains (Fig. 6D). Treatment with 75 µg/mL zinc acetate had no effect on *recA* expression, whereas the combined treatment with AZT and zinc acetate significantly reduced *recA* expression to levels comparable with the DMSO vehicle control treatment (Fig. 6D). These data suggested that AZT interfered with pKpQIL*gfp* conjugation in *K. pneumoniae*, partly by upregulating *recA* expression.

In *K. pneumoniae* Ecl8/pKpQIL*gfp*, some of the most significantly downregulated genes were also associated with the methionine biosynthesis pathway (Fig. 6E). The main end product of methionine biosynthesis is *S*-adenosylmethionine (SAM) (Fig. 6E), a key methyl donor for methylation reactions, which are essential for ribosome biogenesis, DNA/RNA modifications, and many other processes^28^. We hypothesised that since AZT downregulated all the methionine biosynthesis pathway genes, SAM levels would be significantly reduced. Therefore, we tested to see whether the addition of SAM could reverse the AZT-mediated reduction in the CF of pKpQIL*gfp*. The addition of 50, 100 or 125 µg/mL SAM with 0.008 µg/mL AZT significantly increased the CF of pKpQIL*gfp* compared to AZT treatment alone, indicating reversal of the AZT-induced reduction in pKpQIL*gfp* CF (Fig. 6F).

## Discussion

Plasmid-mediated spread of ARGs represents a major threat to global public health. Therefore, targeting plasmid conjugation between bacteria signifies a novel strategy to reduce the dissemination of ARGs. Building on our previous findings of AZT as a plasmid conjugation inhibitor^21^, we screened clinically approved NAs for potential plasmid conjugation-reducing activity in *E. coli* and *K. pneumoniae* using flow cytometry. Through our screening, we identified didanosine, stavudine, and trifluridine as further plasmid conjugation reducing agents. We also found that famciclovir and zalcitabine increased plasmid conjugation in *E. coli*, and aciclovir and valaciclovir increased plasmid transmission in *K. pneumoniae*. As the most promising NA, we explored the mechanisms by which AZT reduced plasmid conjugation in *E. coli* and *K. pneumoniae* using a combination of RNAseq and phenotypic testing.

Our data suggests that aciclovir, famciclovir, valaciclovir, and zalcitabine, commonly used as antiviral agents, could have an unexpected impact on the spread of AMR genes in bacteria. The antibacterial activity of famciclovir has not been explored before, and aciclovir and zalcitabine lack antibacterial activity in *E. coli* and *Salmonella*^29,30^. Based on their reported peak serum concentrations and bioavailability, these NAs may affect the spread of AMR genes in the gut, which is known to be a highly permissive environment for horizontal gene transfer^31^. The mean peak serum concentration of the highest dose of famciclovir (500 mg) is 3.3 µg/mL^32,33^, and we observed an impact at 1 µg/mL, which is lower than the peak serum concentration. For the highest oral dose of zalcitabine (1.5 mg), the peak serum concentration is 0.0252 µg/mL, indicating our results are at concentrations above the peak serum concentration. Furthermore, the bioavailability of both drugs is very high, 77% and greater than 80% for famciclovir and zalcitabine, respectively^33,34^. A 1 g dose of valaciclovir (a pro-drug giving rise to aciclovir) administered orally gives a peak serum concentration of 5–6 µg/mL aciclovir^35^, which is between the 1 and 10 µg/mL concentrations tested in this study. While aciclovir has poor bioavailability (10-20%), valaciclovir has improved bioavailability of 54%^36^. Importantly, these NAs could reach higher concentrations in the gut, where they may affect the gut microbiota and potentiate the spread of AMR genes. A previous study found that a 1.25 mg dose of aciclovir in mice caused gut dysbiosis by reducing the abundance of Bacteroidetes and *Akkermansia muciniphila*^37^. However, further work is necessary to investigate the influence of these NAs on the gut resistome at a population level.

In the mechanistic investigations performed here, aciclovir, famciclovir, valaciclovir, and zalcitabine had no effect on membrane permeability and potential, and ROS generation. However, famciclovir and zalcitabine reduced intracellular ATP content in *E. coli*, and valaciclovir reduced intracellular ATP content in *K. pneumoniae*, following exposure after 4 h. This surprising reduction in intracellular ATP concentrations after exposure to famciclovir, valaciclovir, or zalcitabine raises important mechanistic questions. As these NAs did not affect bacterial growth under the tested concentrations, the decrease in intracellular ATP was unlikely because of generalised metabolic dysfunction or bacterial cell death. A possible explanation is that NAs, after intracellular phosphorylation, could potentially disrupt the balance of the nucleotide pools and replication processes, indirectly affecting central metabolic flux and energy homoeostasis^38,39^. Alternatively, an increased utilisation of the conjugation apparatus may itself cause an energetic burden on the donor cells. Conjugative plasmid transfer is known to require ATP for the assembly and functioning of the type IV secretion system and plasmid DNA processing and translocation^40^. Therefore, increased plasmid conjugation may cause a transient decrease in intracellular ATP concentrations without affecting overall growth kinetics. It is also possible that species-specific differences in nucleotide metabolism or energy regulation^41^ may explain the noted differences in responses between *E. coli* and *K. pneumoniae*. Although our current study does not provide evidence for direct causality between ATP modulation and changes in plasmid conjugation frequency, plasmid acquisition is associated with metabolic burden in bacterial cells^42^. Hence, our results suggest that intracellular energy homoeostasis may provide an additional regulatory mechanism for plasmid transfer.

Several antibiotics have been reported to increase plasmid transfer, typically through induction of cellular stress responses such as the SOS response pathway^43^. The NAs investigated in this study likely differ mechanistically from classical antibiotics. Rather than disrupting cell wall synthesis, translation, or DNA gyrase activity, the NAs presumably interfere with other cellular processes, including DNA replication, motility and cellular metabolism. However, there is evidence that antibiotic-induced plasmid conjugation and SOS response induction can occur through different mechanisms, indicating a lack of correlation between SOS response induction and plasmid transfer^44^. Furthermore, Lopatkin et al.^45^ found that sub-inhibitory concentrations of widely used classes of antibiotics, including aminoglycosides, β-lactams, cephalosporins, macrolides, amphenicols and quinolones did not increase plasmid conjugation frequency, but instead modulate antibiotic-mediated selection, thus promoting and suppressing conjugation frequency. Therefore, while bacterial stress-related pathways may contribute to the increase in plasmid conjugation, our findings suggest that alterations in other cellular mechanisms may represent an additional layer of regulation influencing plasmid transmission.

Through our screening, we found that the NAs didanosine, stavudine, and trifluridine, reduced the conjugation of pCT*gfp* in *E. coli* and pKpQIL*gfp* in *K. pneumoniae* at concentrations that did not impact growth. In agreement with our findings, a previous study reported that 10 µg/mL didanosine did not significantly impact *E. coli* growth across 24 h^46^. A different study found that 20 µM didanosine (~4.7 µg/mL) reduced *K. pneumoniae* growth after 24 h^47^, however, we did not find that 10 µg/mL didanosine affected the generation time or growth kinetics of *K. pneumoniae* Ecl8, which is a different strain than was used by Hind et al.^47^. Stavudine is not antibacterial up to 50 µg/mL after 4 hours^46^, which was within the time-frame of our experiments. There is no reported antibacterial activity of trifluridine in *E. coli* or *K. pneumoniae*, and we did not find that 25 µg/mL trifluridine affected the growth or generation times in either species. Therefore, the effect of didanosine, stavudine, and trifluridine on plasmid conjugation was not due to their antibacterial activity. In both *E. coli* and *K. pneumoniae*, AZT, didanosine, and stavudine had no effect on ROS generation or ATP content. Trifluridine only reduced ATP content in *E. coli*. These four drugs had no impact on membrane permeability or potential, indicating that they reduced plasmid transfer without interfering with membrane physiology. In agreement with our findings, Liu et al. ^48^, found that AZT also had no impact on membrane permeability, potential and ROS generation in *E. coli*. Unlike the NAs that increased plasmid conjugation, trifluridine may reduce pCT*gfp* conjugation by disrupting the availability of ATP needed for the type IV secretion system apparatus. For example, certain fatty acids have been reported to target the ATPase component of type IV secretion systems to inhibit plasmid conjugation^49^, whereas, other compounds have been reported to interfere with energy metabolism to deplete intracellular ATP needed for plasmid conjugation^50,51^. However, trifluridine had no effect on ATP content in *K. pneumoniae*, suggesting that it reduced pKpQIL*gfp* through a different mechanism that remains to be identified.

In *E. coli*, AZT caused downregulation of motility and flagella-associated genes, which was corroborated by the complete inhibition of swimming motility by AZT. When the motility-associated gene *fliE* was inactivated in donor or recipient *E. coli* strains, the result was a reduction of pCT*gfp* conjugation, similar to AZT treatment. Together, these suggested that AZT reduced plasmid conjugation in *E. coli* by inhibiting motility. The link between plasmid transfer and motility has been reported in several studies^52^. In agreement with our findings, Wonterghem et al. ^53^., found that inactivation of the flagella genes *fliF* or *fliK* in donor *E. coli* strains significantly reduced the conjugation of the broad host-range plasmid pKJK10 in liquid broth. As our experiments were also carried out in liquid broth, motility likely plays an important role in facilitating contact between donor and recipient cells. Whether this holds true for plasmid conjugation on solid surfaces remains to be fully established, but a previous study reported that the absence of motility resulted in *Xanthomonas retroflexus* and *Pseudomonas putida* enhanced plasmid uptake in biofilms^54^. In addition to chromosomal genes, type IV pilus (HXG14_RS00465, *pilP*, and *pilV*) genes in pCT*gfp* were also significantly downregulated in pKpQIL*gfp* (Supplementary Data 1). The type IV pilus is critical for facilitating mating pair stabilization between donor and recipient cells^55^. Previously, loss of *pilV* was shown to impair plasmid conjugation in liquid broth^56^. Therefore, AZT-induced reduction in motility coupled with decreased pili production likely reduces donor and recipient contact and prevents mating pair stabilisation in liquid broth, thereby reducing plasmid transfer. Another layer of complexity is the interplay between the loss of motility, reduced plasmid conjugation, and intracellular energy availability, which remains to be established. Whilst plasmid conjugation requires ATP for the assembly of the type IV secretion system and translocation of plasmid DNA, flagellar rotation is driven by the proton motive force. Hence, future studies aimed at elucidating the relationship between the energy costs of flagellar rotation and plasmid conjugation apparatus will be needed to clarify the relationship between energy balance, motility, and plasmid conjugation.

In *K. pneumoniae*, AZT caused upregulation of DNA damage and SOS response genes. The addition of zinc acetate restored the transfer of pKpQIL*gfp* in the presence of AZT, which was attributed to a reduction in *recA* expression. This is in agreement with previous studies, which have demonstrated the ability of zinc acetate to inhibit RecA activity^57,58^. The ability of zinc acetate to restore pKpQIL*gfp* transfer in AZT-treated *K. pneumoniae* may also be attributed to its ability to disrupt interactions between single-stranded DNA and RecA, as previously observed^58^. Therefore, increased *recA* expression caused by AZT may encourage such interactions and, as a result, SOS-mediated degradation of the plasmid transferred to the recipient cells. We also found that AZT downregulated methionine biosynthesis pathway genes in *K. pneumoniae*, and likely SAM biosynthesis as the endpoint product. In line with this hypothesis, exogenous addition of SAM restored the conjugation of pKpQIL*gfp* in the presence of AZT. A previous study hypothesised that plasmid methylation by SAM may protect against restriction activity in recipient *K. pneumoniae*. Oo et al.^59^ identified a link between spermidine synthesis and plasmid conjugation, with *K. pneumoniae* mutants of the spermidine synthesis pathways displaying lower conjugation frequencies. The overexpression of *metK* in the spermidine synthesis mutant strains caused a significant increase in plasmid conjugation^59^. Therefore, AZT treatment may reduce the protective effect of methylation by SAM, leading to the observed reduction in pKpQIL*gfp* conjugation. Such protective effects of SAM have been formerly described in the context where the donor and recipient possess similar restriction modification systems.

Although our work provides an important mechanistic insight into how NAs may modulate plasmid transfer, a limitation was that all experiments were carried out in vitro. Whilst this allowed us to explore the mechanistic effects of NAs on plasmid conjugation in a controlled and reproducible manner, it does not capture the complexity of plasmid dynamics in natural environments. In particular, plasmid conjugation within the gastrointestinal tract is influenced by multiple factors, including host factors, microbial community interactions, and nutrient availability^60^. Therefore, future work is necessary to determine whether NAs affect plasmid transfer in the same manner in physiologically relevant systems, such as ex vivo faecal models or in vivo animal infection models^61^. Another limitation of our study was the use of two AMR plasmids in two bacterial hosts, which may limit the general applicability of NAs as plasmid conjugation reducing agents. Hence, the effect of NAs on plasmid transmission needs to be studied in other plasmid types and bacterial hosts. Nevertheless, defining these mechanisms under controlled conditions using two different clinically important AMR plasmids represents an important first step toward understanding how clinically approved compounds may influence horizontal gene transfer.

In summary, our results indicated that additional clinically approved NAs didanosine, stavudine, and trifluridine possess plasmid conjugation reducing activities in *E. coli* and *K. pneumoniae*. Additionally, certain NAs increased plasmid conjugation, suggesting their role as potential promoters of plasmid dissemination. Our mechanistic investigation revealed that AZT reduced plasmid conjugation in *E. coli* by interfering with motility gene expression, corroborating the reduced plasmid transfer observed upon genetic inactivation of motility. In *K. pneumoniae*, AZT reduced plasmid conjugation by inducing DNA damage and SOS response genes and suppressing methionine biosynthesis and metabolism. That this inhibition can be restored by targeting RecA with zinc acetate or by supplementing *S*-adenosyl-methionine underscores the important contribution of these pathways and provides a mechanistic understanding of how AZT reduces plasmid dissemination. Therefore, AZT could provide a structural scaffold for the development and optimisation of more potent conjugation inhibitors.

## Methods

### Bacterial strains and growth conditions

The bacterial strains used are listed in Table 1. Unless stated otherwise, all strains were grown in Luria–Bertani (LB) broth (Sigma-Aldrich, USA) and incubated at 37 °C with aeration.

**Table 1:** List of bacterial strains used in this study

Strain ID	Description	Reference
EC24	*Escherichia coli* EC958, cured of pEC958, carrying IncK plasmid pCT*gfp* (*gfp-aph* inserted into the *bla*_CTXM-14_ gene)	^21^
EC25	*Escherichia coli* EC958, cured of pEC958, with chromosomal *mCherry* (*mCherry-aph* cassette inserted between *putPA* on the chromosome)	^21^
EC83	EC24 with the *fliE* gene inactivated by insertion of the hygromycin resistance gene *hph*	This study
EC84	EC25 with the *fliE* gene inactivated by insertion of the hygromycin resistance gene *hph*	This study
KP18	*Klebsiella pneumoniae* Ecl8 with chromosomal *mCherry* (*mCherry-aph* cassette inserted between *putPA* on the chromosome)	^21^
KP19	*Klebsiella pneumoniae* Ecl8 carrying IncF plasmid pKpQIL*gfp* (*gfp-aph* cassette inserted into the *bla*_KPC-3_ gene on pKpQIL)	^21^
SA01	*Staphylococcus aureus* ATCC 29213	ATCC
PA02	*Pseudomonas aeruginosa* ATCC 27853	ATCC

### Generation of *fliE*-inactivated *Escherichia coli* strains

The *fliE* gene, encoding the flagellar hook-basal body complex protein, was inactivated in strains EC24 and EC25 (Table 1) by inserting the *hph* gene, encoding for hygromycin B phosphotransferase, using the pACBSCE plasmid^62^. Firstly, the *hph* gene was amplified from the pSIM18 plasmid^63^ using primers (Supplementary Table 2) that have flanking 40 bp homology to the *fliE* gene in *E. coli* EC958. The arabinose-inducible recombineering plasmid pACBSCE was electroporated into EC24 and EC25 with subsequent electroporation of the PCR-amplified hygromycin resistance cassette. Successful recombinants were selected on LB agar supplemented with 150 µg/mL hygromycin (Sigma-Aldrich, USA). PCR and Sanger sequencing (Eurofins Genomics, UK) using primers (Supplementary Table 2) that bind upstream and downstream of *fliE*, were used to verify the successful insertion of the *hph* gene.

### Antimicrobial susceptibility testing

To determine suitable concentrations of nucleoside analogues and selected antibiotics, minimum inhibitory concentrations (MICs) were determined by the broth microdilution method^64^. Nucleoside analogues were purchased from Sigma-Aldrich (USA) or Cambridge Bioscience (UK). The MICs were recorded as the lowest concentration at which no visible growth was observed.

### Measuring plasmid transmission and conjugation using flow cytometry

The transmission of pCT*gfp* in *E. coli* EC958 and pKpQIL*gfp* in *K. pneumoniae* Ecl8 in the presence of the nucleoside analogues was assessed by flow cytometry as described previously^21^. Briefly, 1 mL of overnight cultures of donor (*E. coli* EC958/pCT*gfp* or *K. pneumoniae* Ecl8/pKpQIL*gfp*) and recipient (*E. coli* EC958 or *K. pneumoniae* Ecl8 with chromosomal *mCherry*) strains were pelleted, washed in sterile PBS, and adjusted to an OD_600_ of 0.5. Equal volumes were mixed (1:1 ratio), and 20 µL of the mix was added to 180 µL LB broth with either the test compound or DMSO (vehicle control) in a 96-well round-bottom plate (Corning, USA). Plates were incubated at 37 °C with gentle agitation (100 rpm) for 4 h. After incubation, 20 µL was serially diluted 1:1000 in filter-sterilised Dulbecco’s PBS (Sigma-Aldrich, USA) and analysed on an Attune NxT flow cytometer with an Autosampler (Thermo Scientific, USA). GFP and mCherry emissions were detected using BL1-H and YL2-H channels, respectively. For each sample, 10,000 bacterial events were recorded. Conjugation was assessed by quantifying GFP+ (donor), mCherry+ (recipient), and GFP + /mCherry+ (transconjugant) populations using previously described gating strategies^21^. In the initial screening of NAs, plasmid transmission as a percentage of control was calculated as the number of transconjugants in the treated sample divided by the number of transconjugants in the control sample, multiplied by 100. In all the follow-up experiments with selected NAs, plasmid conjugation frequency was calculated as the number of transconjugant cells divided by the number of recipient cells, unless otherwise specified.

### Bacterial motility assays

Bacterial motility was assessed by swimming assays. Plates were prepared with 0.3% Difco granulated agar (BD, USA, cat. no. 214530), as previously described^65^, and allowed to dry overnight at room temperature. These were then inoculated by stabbing with overnight cultures adjusted to an OD_600_ of 0.5 and incubated at 37 °C for 18 h. Swimming motility was determined by measuring the diameter and calculating the area of the swimming zone. Data presented are the means ± standard deviation of three independent experiments, each consisting of three biological replicates.

### Measurement of bacterial membrane potential

The impact of nucleoside analogues on *E. coli* and *K. pneumoniae* membrane potential was measured as previously described^66^. All media, plates, and the plate reader were warmed to 37 °C prior to use. Briefly, overnight bacterial cultures were subcultured in 5 mL LB broth (1% inoculum), grown to the exponential phase, and adjusted to an OD_600_ of 0.5 in LB broth supplemented with 0.5 mg/mL bovine serum albumin. The cells were then dispensed into black polystyrene 96-well plates (Greiner Bio-One, Austria) and the autofluorescence of bacterial cells was measured for 5 min. DiSC_3_(5) (3,3’-dipropylthiadicarbocyanine iodide) (Cayman Chemical, USA) was dissolved in DMSO and added to the cells at a final concentration of 1 µM. The fluorescence quenching of DiSC_3_(5) was monitored for 15 min before the addition of test compounds. As positive control, 20 µg/mL polymyxin B was used as previously described^66^. Fluorometric measurements were recorded every minute using a BioTek Synergy H1 Multimode Reader (Agilent Technologies, USA), with vigorous shaking between measurements, at excitation and emission wavelengths of 610 nm (±10) and 660 nm (±10), respectively. To ensure that the nucleoside analogues did not interfere with DiSC3(5) fluorescence, the assay was also carried out without bacterial cells. Data presented are the means ± standard deviation of three biological replicates, each tested on independent occasions.

### Measurement of reactive oxygen species generation

The effect of nucleoside analogues on reactive oxygen species (ROS) production in *E. coli* and *K. pneumoniae* cells was determined using the ROS-Glo H_2_O_2_ Assay Kit (Promega, USA) as per the manufacturer’s instructions. Briefly, overnight cultures were diluted to an OD_600_ of 0.05 in LB broth, and 80 µL was dispensed into white, flat-bottom 96-well plates (Corning, USA). A 20 µL volume of H_2_O_2_ substrate solution combined with the nucleoside analogues, DMSO vehicle control, or 50 µM menadione as positive control, was added to bacterial cells, and incubated for 4 h at 37 °C with shaking (100 rpm). Following incubation, 100 µL of ROS-Glo Detection Solution was added to each well, and the plate was incubated at room temperature for 20 min. Luminescence was then measured using a BioTek Synergy H1 Multimode Reader (Agilent Technologies, USA). Data presented are the mean ± of three biological replicates tested on three independent occasions and expressed as luminescence relative to DMSO vehicle control.

### Measurement of intracellular ATP content

The effect of nucleoside analogues on ATP production in *E. coli* and *K. pneumoniae* cells was determined using the BacTiter-Glo Kit (Promega, USA) as per the manufacturer’s instructions. Briefly, overnight bacterial cultures were diluted to an OD_600_ of 0.5 in LB broth, and 10 µL of this solution was mixed with 90 µL of LB broth supplemented with nucleoside analogues, DMSO vehicle control, or 2 µg/mL doripenem as a positive control, in white, flat-bottom 96-well plates. Bacterial cells were incubated for 4 h at 37 °C with shaking (100 rpm), and then equilibrated to room temperature for 5 min. A 100 µL volume of BacTiter-Glo Reagent was added to each well, and the contents of the plate were mixed briefly on an orbital shaker for 5 min. Luminescence was then measured using a BioTek Synergy H1 Multimode Reader (Agilent Technologies, USA). Data presented are the mean ± standard deviation of three biological replicates tested on three independent occasions and expressed as luminescence relative to DMSO vehicle control.

### Measurement of inner-membrane and outer-membrane permeability

The effect of nucleoside analogues on inner- and outer-membrane permeability in *E. coli* and *K. pneumoniae* cells was determined using propidium iodide (PI) and *N*-phenyl-1-naphthylamine (NPN) uptake, respectively. Stock solutions of NPN (Fisher Scientific, USA) and PI (Sigma-Aldrich, USA) were prepared in ethanol and sterile distilled water, respectively, and diluted to working stock solutions in 5 mM HEPES buffer. Overnight bacterial cultures were subcultured in 5 mL LB broth (1% inoculum), grown to the exponential phase, and then adjusted to an OD_600_ of 0.5 in 5 mM HEPES buffer. A 198 µL volume of bacterial suspension was mixed with 1 µL of 100 µM NPN or PI (10 µM final concentration) and 1 µL of nucleoside analogues, DMSO vehicle control, or sodium dodecyl sulphate as a positive control, in black, flat-bottom 96-well plates (Greiner Bio-One, Austria). The plates were incubated in darkness for 30 minutes at room temperature. Fluorescence was measured using a BioTek Synergy H1 Multimode Reader (Agilent Technologies, USA). For NPN, excitation and emission wavelengths were 350 nm and 420 nm, and for PI, the excitation and emission wavelengths were 540 nm and 640 nm. Data presented are the mean ± of three biological replicates tested on three independent occasions and expressed as NPN or PI fluorescence relative to DMSO vehicle control.

### RNA extractions

Four overnight cultures of EC24 (*E. coli* EC958/pCT*gfp* and KP19 (*K. pneumoniae* Ecl8/pKpQIL*gfp* were subcultured in 10 mL LB broth (2% inoculum) supplemented with 0.008 µg/mL AZT or an equal volume of DMSO as a vehicle control. Bacterial cells were grown to the exponential phase (OD_600_ of 0.5) at 37 °C with aeration, and total RNA was extracted using the Monarch Total RNA Miniprep Kit (NEB, USA), with on-column DNase I treatment to eliminate genomic DNA contamination as per the manufacturer’s instructions. RNA quality and quantity were determined using a NanoDrop spectrophotometer (Thermo Scientific, USA) and the Qubit RNA Broad Range Assay Kit (Invitrogen, USA), respectively.

### RNA sequencing and data analysis

Library preparation, sequencing and bioinformatic data analysis were carried out by GeneWiz UK Ltd. rRNA depletion was performed using a NEBNext rRNA Depletion Kit (Bacteria). Library preparation was performed using a NEBNext Ultra II RNA Library Prep Kit (NEB, USA) for Illumina according to the manufacturer’s instructions. Briefly, enriched RNAs were fragmented according to the manufacturer’s instructions. First-strand and second-strand cDNA were subsequently synthesised. cDNA fragments were end-repaired and adenylated at 3’ ends, and a universal adapter was ligated to cDNA fragments, followed by index addition and library enrichment with limited-cycle PCR. Sequencing libraries were validated using the NGS Kit on the Agilent 5300 Fragment Analyzer (Agilent Technologies, USA) and quantified by using a Qubit 4.0 Fluorometer (Invitrogen, USA). The sequencing libraries were multiplexed and loaded on the flow cell of an Illumina NovaSeq 6000 instrument according to the manufacturer’s instructions. The samples were sequenced using a 2 × 150 Pair-End (PE) configuration v1.5. Image analysis and base calling were conducted by the NovaSeq Control Software v1.7 on the NovaSeq instrument. Raw sequence data (.bcl files) generated from Illumina NovaSeq were converted into fastq files and de-multiplexed using the Illumina bcl2fastq program version 2.20. One mismatch was allowed for index sequence identification. After investigating the quality of the raw data, sequence reads were trimmed to remove possible adapter sequences and nucleotides with poor quality using Trimmomatic v.0.36. The trimmed reads were mapped to the reference genomes using the Bowtie2 aligner v.2.2.6 to generate BAM files. Unique gene hit counts were calculated using featureCounts from the Subread package v.1.5.2. Only unique reads that fell within gene regions were counted. After the extraction of gene hit counts, the gene hit counts table was used for downstream differential expression analysis. Using DESeq2, a comparison of gene expression between DMSO- and AZT-treated samples was performed. The Wald test was used to generate p-values and log_2_ fold-changes. Genes with an adjusted p value ≤ 0.05 and absolute log_2_ fold-change > 0.5 were called as differentially expressed genes for each comparison.

### Ethics

No ethical approval was required for this work.

## Supplementary information

Supplementary Figures and Tables.

Supplementary Data 1.

## Data Availability

The RNA sequencing data generated in this project have been deposited in ArrayExpress under accession code E-MTAB-15827.
