# Supplementary material for: Mechanistic insights into plasmid transfer inhibition in Enterobacterales by nucleoside analogues
Source: NPJ Antimicrob Resist. 2026 Apr 14;4:23. doi: 10.1038/s44259-026-00197-5 (PMC13079834; doi:10.1038/s44259-026-00197-5)
Supplement: Supplementary file 1 — Supplementary Figures and Tables. [file 44259_2026_197_MOESM1_ESM.docx]

**Supplementary figures and tables**

**Mechanistic insights into plasmid transfer inhibition in Enterobacterales by nucleoside analogues**

Ilyas Alav^1,2^, Ayesha Ashraf^1^, Parisa Pordelkhaki^1^, and Michelle M. C. Buckner^1#^

^1^Department of Microbes, Infection and Microbiomes, School of Infection, Inflammation and Immunology, College of Medicine and Health, University of Birmingham, Birmingham, UK

^2^Current address: Sir William Dunn School of Pathology, University of Oxford, Oxford, UK.

^#^Corresponding author

Email: [m.buckner@bham.ac.uk](mailto:m.buckner@bham.ac.uk)

**Supplementary Table 1.** Susceptibility of *Escherichia coli* EC958 and *Klebsiella pneumoniae* Ecl8 strains to nucleoside analogues.

| **Strain** | *E. coli* EC958 with pCT*gfp* | *E. coli* EC958 *mCherry* | *K. pneumoniae* Ecl8 with pKpQIL*gfp* | *K. pneumoniae* Ecl8 *mCherry* |
| --- | --- | --- | --- | --- |
| **Nucleoside analogue** | **Minimum inhibitory concentration (µg/mL)** | | | |
| Aciclovir | >256 | >256 | >256 | >256 |
| Azidothymidine | 2 | 2 | 2 | 2 |
| Didanosine | 128 | 128 | 128 | 128 |
| Emtricitabine | >256 | >256 | >256 | >256 |
| Famciclovir | >256 | >256 | >256 | >256 |
| Ganciclovir | >256 | >256 | >256 | >256 |
| Idoxuridine | >256 | >256 | >256 | >256 |
| Lamivudine | >256 | >256 | >256 | >256 |
| Penciclovir | >256 | >256 | >256 | >256 |
| Stavudine | >256 | >256 | >256 | >256 |
| Telbivudine | >256 | >256 | >256 | >256 |
| Trifluridine | >256 | >256 | >256 | >256 |
| Valaciclovir | >256 | >256 | >256 | >256 |
| Vidarabine | >256 | >256 | >256 | >256 |
| Zalcitabine | >256 | >256 | >256 | >256 |


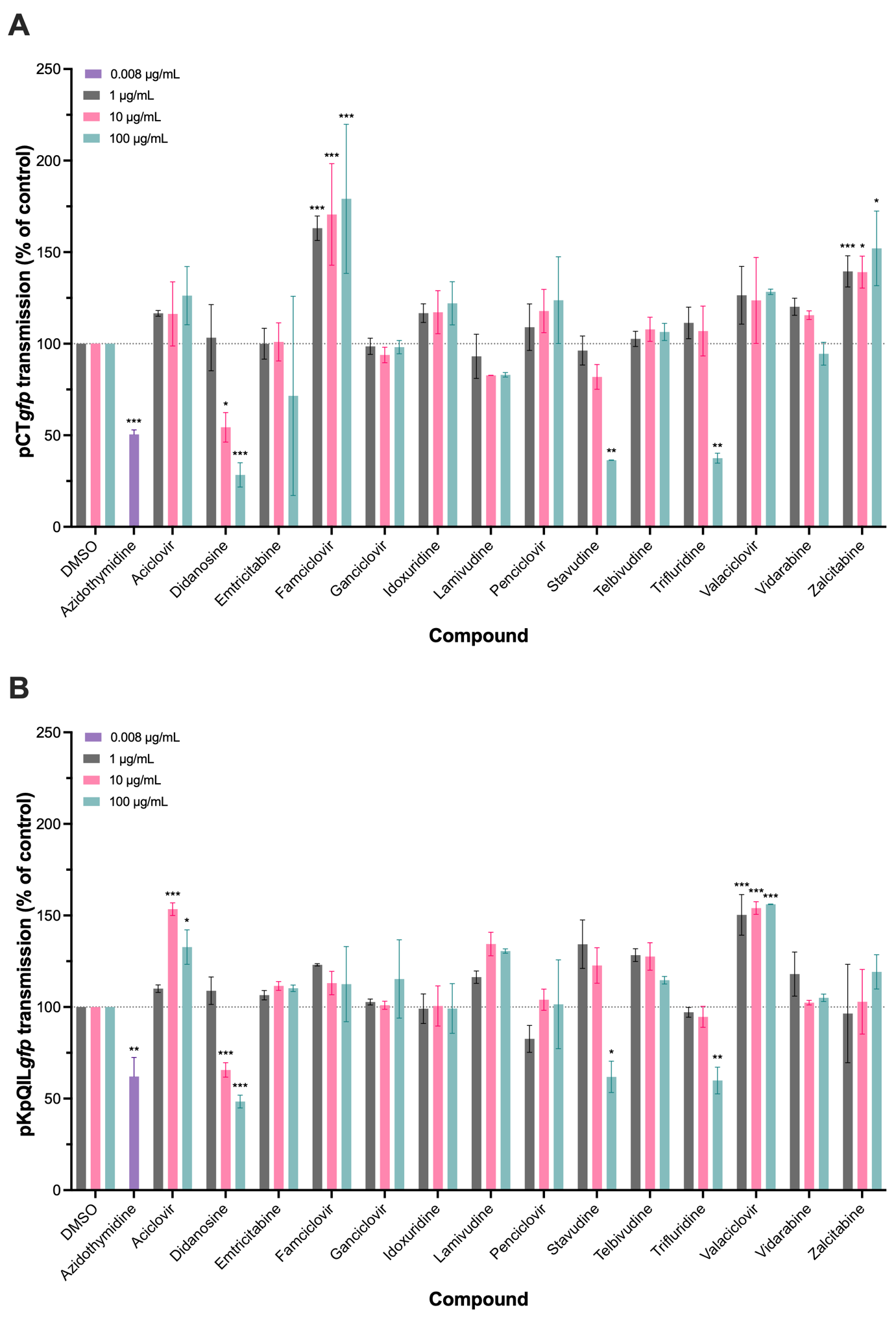


**Supplementary Figure 2. Flow cytometry-based screening of clinically approved nucleoside analogues (NAs) for plasmid transmission inhibition activity.** The impact of NAs at 1, 10 and 100 µg/mL, or 0.008 µg/mL azidothymidine on the transmission of **A)** pCT*gfp* from EC24 (*E. coli* EC958c carrying pCT*gfp*) to the recipient EC25 (*E. coli* EC958c *mCherry*) and **B)** pKpQIL*gfp* from KP19 (*K. pneumoniae* Ecl8 carrying pKpQIL*gfp*) to the recipient KP18 (*K. pneumoniae* Ecl8 *mCherry*). Briefly, the donor and recipient strains were mixed at a 1:1 ratio and then added to LB broth supplemented with the indicated concentration of NAs or an equal volume of DMSO as vehicle control. Conjugation assays were carried out for 4 h at 37 °C with gentle agitation (100 r.p.m). Plasmid transmission was determined as the number of transconjugants treated with NAs relative to the number of transconjugants treated with DMSO control. Data presented are the mean ± standard deviation of two independent experiments, each consisting of four biological replicates. The transmission of plasmids treated with DMSO were compared to those treated with the NAs using one-way ANOVA, followed by the Dunnett’s test to correct for multiple comparisons. Significantly different results are indicated with * (*P*≤0.05) ** (*P*≤0.01) or *** (*P<*0.001).


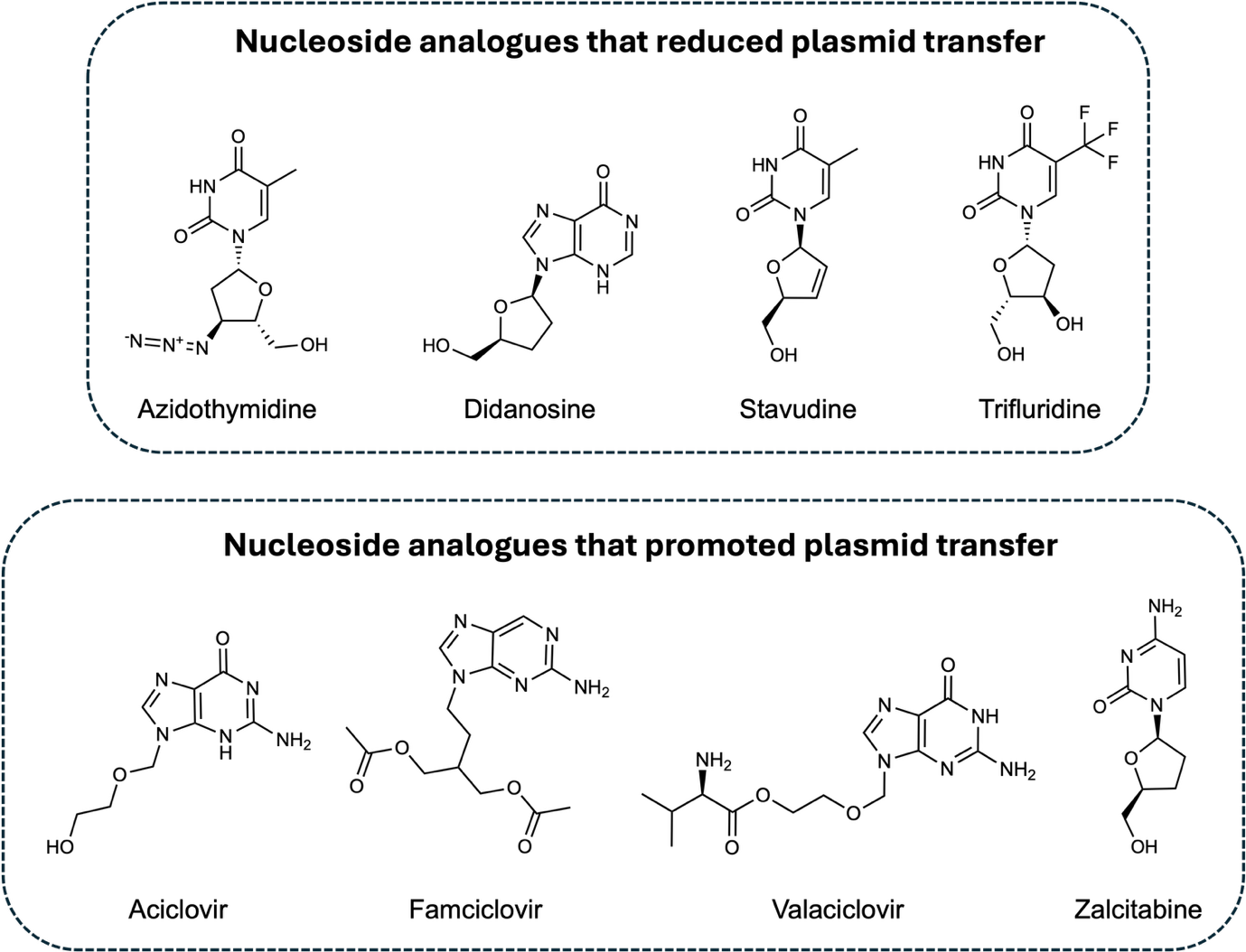


**Supplementary Figure 3. The chemical structures of nucleoside analogues (NAs) that reduced or promoted plasmid transfer.** The NAs that reduced plasmid transfer were adenosine (didanosine) or thymidine (azidothymidine and stavudine) analogues, with trifluridine being a deoxyuridine analogue. The NAs that promoted plasmid transfer were guanosine (aciclovir and valaciclovir) or cytosine (zalcitabine) analogues.


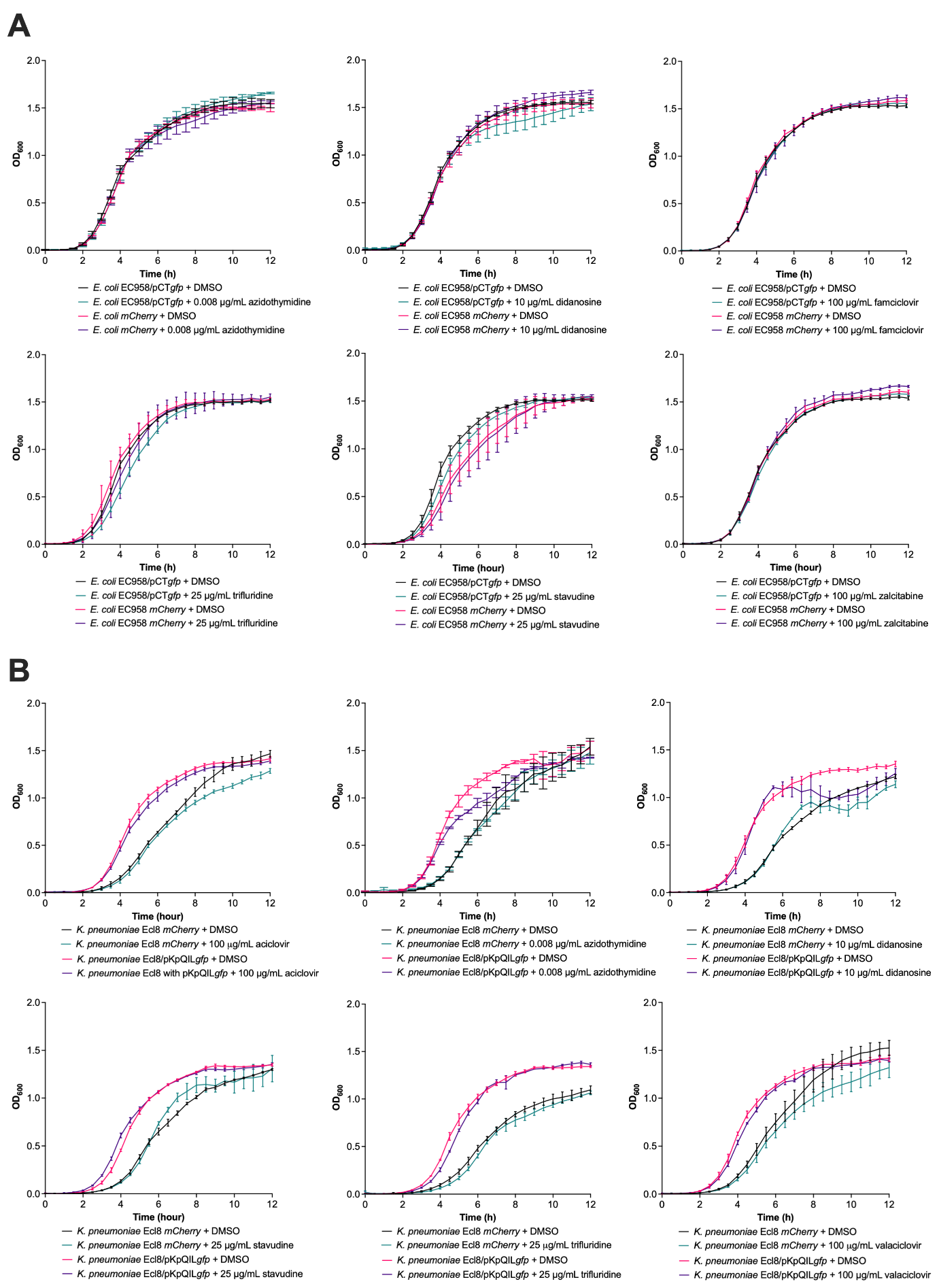


**Supplementary Figure 4. The growth of *Escherichia coli* and *Klebsiella pneumoniae* strains in the presence of selected nucleoside analogue concentrations.** The growth kinetics **A)** of *E. coli* EC958/pCT*gfp* and *E. coli* EC958 *mCherry* and **B)** *K. pneumoniae* Ecl8 *mCherry* and *K. pneumoniae* Ecl8/pKpQIL*gfp*, in the presence of LB broth supplemented with nucleoside analogues or an equal volume of DMSO as solvent control. Growth was measured at 37 °C with shaking (150 r.p.m), with OD_600_ readings taken at 30-minute intervals over 12 hours. Data presented are the mean ± standard deviation of three independent experiments, each consisting of three biological replicates.

**
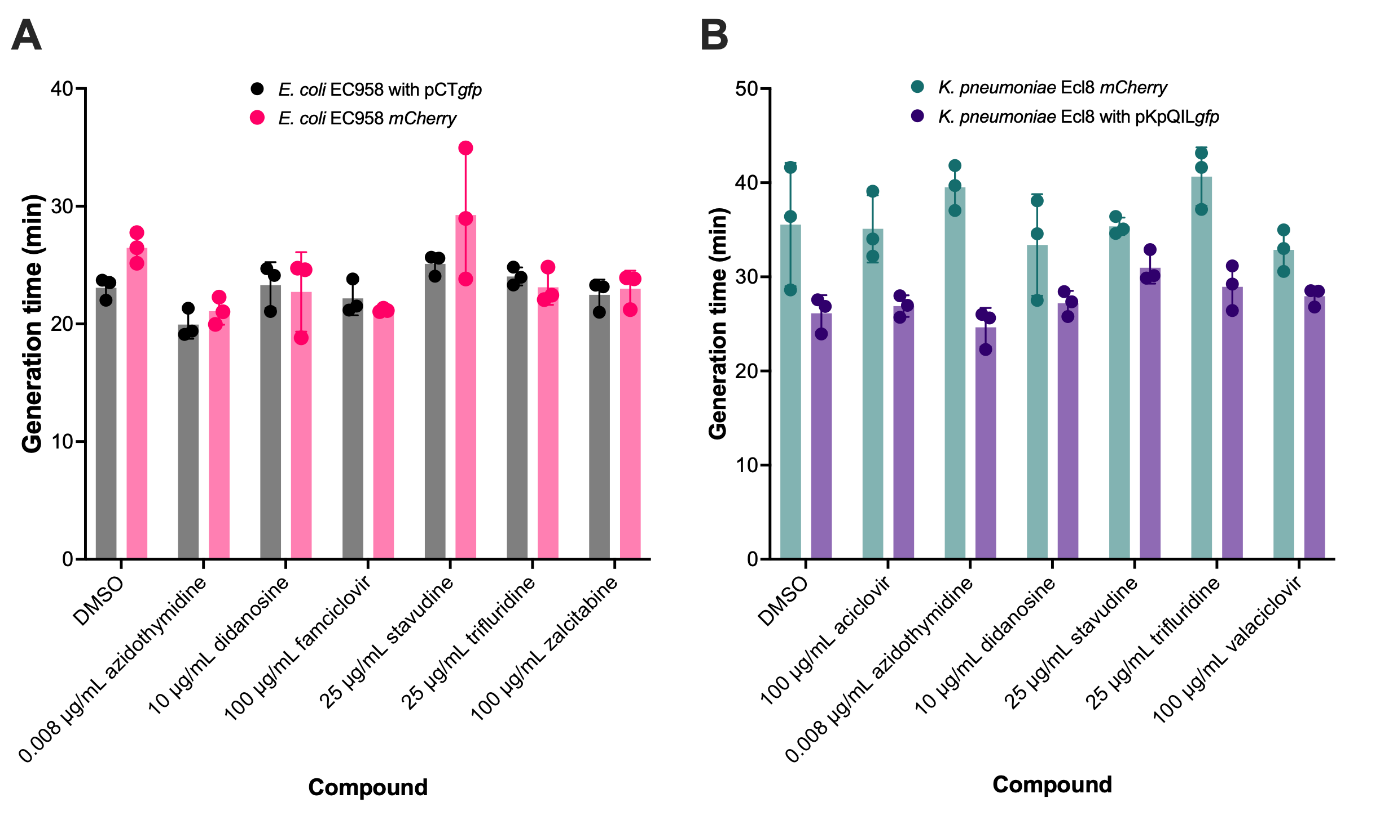
 Supplementary Figure 5. The impact of the selected nucleoside analogue concentrations on the generation times of *Escherichia coli* and *Klebsiella pneumoniae* strains.** The mean generation times of **A)** *E. coli* EC958 *mCherry* and *E. coli* EC958 pCT*gfp* and **B)** *K. pneumoniae* Ecl8 *mCherry* and *K. pneumoniae* Ecl8 with pKpQIL*gfp*, grown in LB broth supplemented with nucleoside analogues or an equal volume of DMSO as vehicle control. Data presented are the mean ± standard deviation of three independent experiments, each consisting of three biological replicates. The mean generation time of each strain grown in DMSO was compared to the mean generation times grown in nucleoside analogues using one-way ANOVA, followed by the Dunnett’s test to correct for multiple comparisons. There were no significant differences.


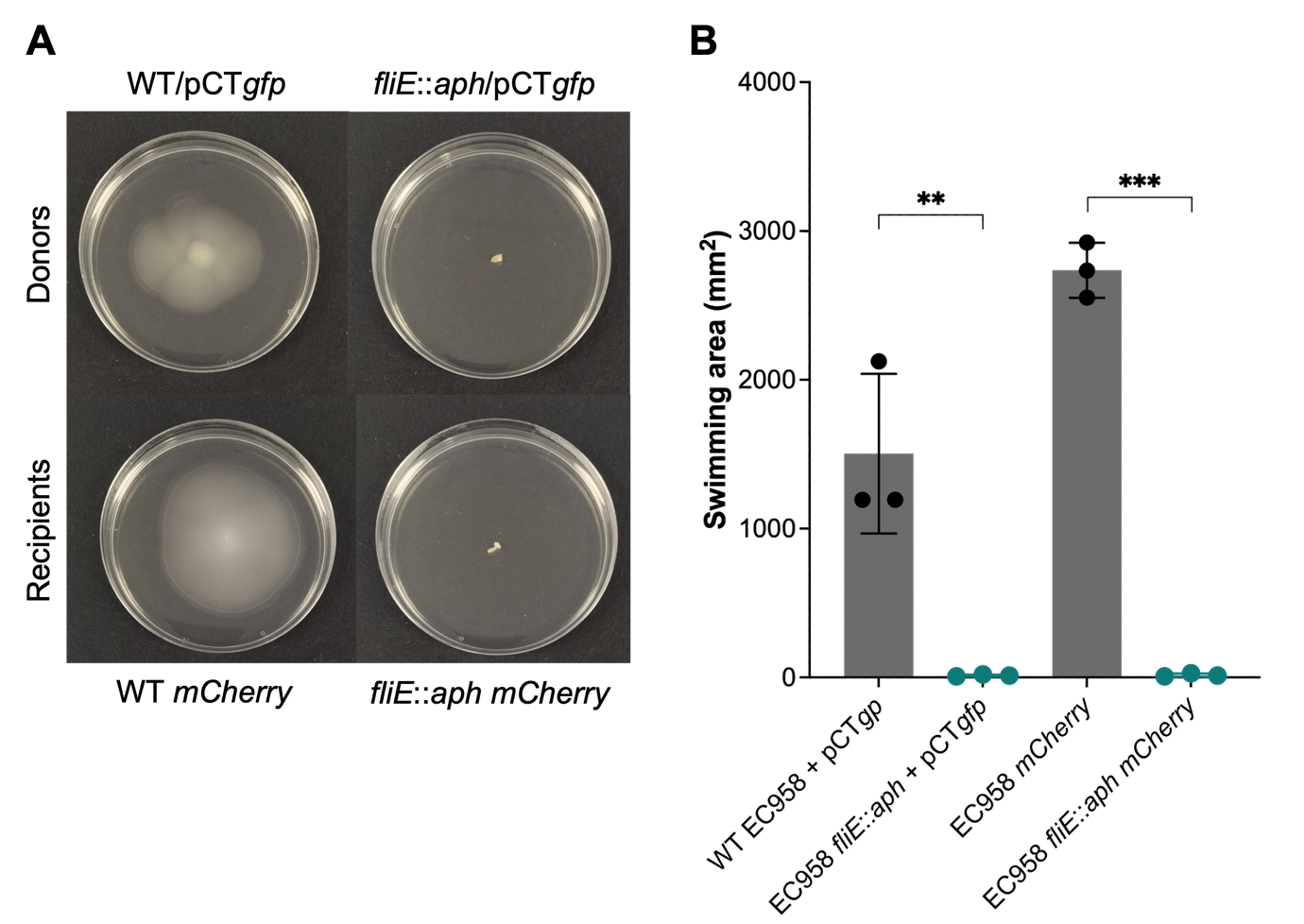
 **Supplementary Figure 6. Swimming motility of EC958 carrying pCT*gfp* and EC958 *mCherry*, and their *fliE* inactivated mutant strains.** Swimming motility was measured using 0.3% agar and inoculated by stabbing prepared culture into the medium. Plates were incubated at 37 °C for 18 h. Swimming motility was determined by measuring the diameter and calculating the area of the swimming zone. **A)** Representative images of swimming motility by wild-type EC958 carrying pCT*gfp* and EC958 *mCherry*, and their *fliE* inactivated mutant strains. **B)** Swimming area of wild-type EC958 carrying pCT*gfp* and EC958 *mCherry*, and their *fliE* inactivated mutant strains. Data presented are the mean ± standard deviation of three independent experiments, each consisting of three biological replicates. The mean swimming area of the *fliE* inactivated mutant strains were compared to their wild-type parent strains using unpaired two-tailed *t*-test. Significantly different results are indicated with ** (*P*≤0.01) or *** (*P<*0.001).

**Supplementary Table 2.** Primers used for the generation and sequencing of the *fliE* inactive mutant *E. coli* EC958 strains

| **Primer ID** | **Description** | **Sequence (5’-3’)** |
| --- | --- | --- |
| P244 | Forward primer for amplifying the hygromycin resistance gene from pSIM18 with flanking sites that have homology to the *fliE* gene in *E. coli* EC958 | TGCTATTTAGCGCCTTTGTCTTATTGACTTACTGGTAGGCTCATTCAAATATGTATCCGCTC |
| P245 | Reverse primer for amplifying the hygromycin resistance gene from pSIM18 with flanking sites that have homology to the *fliE* gene in *E. coli* EC958 | GGATCGGATATAATTAAACAAGCATAGTCAACAGGTTACACCTATTCCTTTGCCCTCGGACG |
| P246 | Forward primer that binds downstream of the *fliE* gene in *E. coli* EC958 | CACGCTGTGGGATCGGATA |
| P247 | Reverse primer that binds upstream of the *fliE* gene in *E. coli* EC958 | TGACGCTCCCATTATTTGTGC |

**Supplementary Data 1.** Lists of significant differentially expressed genes (DEGs) in *Escherichia coli* EC958 with pCT*gfp* and *Klebsiella pneumoniae Ecl8 with pKpQIL*gfp treated with 0.008 µg/mL azidothymidine compared to DMSO control.
